# 

**Published:** 1824-09-01

**Authors:** 


### Contents

OF

No. 2. Vol. I. (NEW SERIES) for SEPTEMBER, 1824.

[*No.* 18, *ANALYTICAL SERIES.*]

Art. I.

LALLEMAND ON THE BRAIN.

Recherches Anatomico-Pathologiques sur l'Encephale, &c. or Anatomico-Pathological Researches on the Brain and its Appendages. By Professor Lallemand . .. .. .. 265

II.

EDINBURGH MEDICO-CHIRURGICAL TRANSACTIONS.

I. Dr. Kellie's Cases and Dissections, with Reflections on the Pathology of the Brain .. .. .. .. .. .. .. 285
II. Dr. Coombe's Case of Anemia .. .. .. .. .. .. 295
III. Case of Hydrocephalus, with Bifid Brain, by Dr. Duncan, Jun. .. .. .. .. .. .. .. .. .. .. .. 297
IV. Remarkable Case of Phrenitis, by Mr. Rhind .. .. 299
V. Dr. Hay's Case of Dysphagia, &c. .. .. .. .. 300
VI. Dr. Holmes' Case of Malformation of the Heart .. .. 301
VII. Dr. Moncrief's Case of Tuberculated Peritoneum .. 302

III.

SPINAL DISEASES.

1. Mr. Shaw's Engravings illustrative of the Nature and Treatment of Distortions of the Spine .. .. .. .. } 303
2. Dr. Jarrold's Enquiry into the Causes of Curvature of the Spine &c. &c. .. .. .. .. .. .. .. .. .. }

IV.

TOXICOLOGY.

Dr. Paris on Poisons. [Medical Jurisprudence.] .. .. .. 316

V.

Drs. ELLIOTSON, DICKSON, and MARTINET on Quinine and Sulphate of Quinine .. .. .. .. .. .. .. .. 331

VI.

INFANTICIDE.

Dr. PARIS, Dr. BECK, Dr. SMITH, Dr. MALE and others, on Infanticide .. .. .. .. .. .. .. .. .. .. 337

VII.

TRANSACTIONS OF THE KING'S AND QUEEN'S COLLEGE OF PHYSICIANS IN IRELAND. VOL. IV.

I. Observations on a Species of Premature Labour .. .. 355
II. Dr. J. CRAMPTON on Tinea .. .. .. .. .. .. 359
III. ———— on the fatal Result of Mercurial Ointment 360
IV. ———— Case of Chorea .. .. .. .. .. 363
V. Dr. EVANSON'S Cases of Hydrocephalic Fever .. .. 364
VI. Dr. CLARKE on Variola post Vaccinam .. .. .. 366
VII. Dr. PICKELLS' Case of a Woman who discharges Insects from the Stomach .. .. .. .. .. .. .. .. 367
VIII. Mr. ADAMS' Operations on the Neck .. .. .. .. 371

VIII.

PHLEGMATIA DOLENS.

1. Dr. DAVIS on the Proximate Cause of Phlegmatia Dolens .
2. Dr. FRANCIS on Phlegmatia Dolens .. .. .. .. ..
3. Dr. HOSACK on Phlegmatia Dolens .. .. .. .. ..
} 378

IX.

Dr. SHEARMAN on Chronic Debility .. .. .. .. .. 391

X.

CATARACT.

1. Mr. GUTHRIE on Cataract .. .. .. .. .. ..
2. Mr. STEVENSON on Cataract .. .. .. .. .. ..
3. Dr. BOWEN on Cataract .. .. .. .. .. ..
} 396

XI.

Mr. HEY, Jun. on Puerperal Fever .. .. .. .. .. 414

### XII.

### Quarterly Periscope OF PRACTICAL MEDICINE; BEING *THE SPIRIT OF THE MEDICAL JOURNALS,* FOREIGN AND DOMESTIC.

### I. PHYSIOLOGY.

1. Physiological Metamorphoses .. .. .. .. .. .. 436

2. Physiology of the Nerves .. .. .. .. .. .. .. 437

3. Temporary Blindness .. .. .. .. .. .. .. .. 438

4. Occlusion of Intestinal and Urinary Passages .. .. .. 438

5. Theory of Fever .. .. .. .. .. .. .. .. .. 439

6. Magendie's Physiological Experiments .. .. .. .. 440

### II. PATHOLOGY.

1. Paralysis on the same Side as the Injury .. .. .. .. 446

2. Dr. Martland on Melena .. .. .. .. .. .. .. 450

3. On Organic Changes in the Bronchia .. .. .. .. .. 451

4. On Spinal Consumption .. .. .. .. .. .. .. 456

5. Arachnitis .. .. .. .. .. .. .. .. .. .. 458

6. On the Physiology and Pathology of the Spleen .. .. .. 460

7. On Thickening of the Coats of the Stomach .. .. .. .. 462

### III. SURGERY.

1. Injuries of the Head, Concussion, Compression &c. .. .. 464

2. Extirpation of the Thyroid Gland .. .. .. .. .. 472

3. Dr. Civiale's Lithontriptor, or Stone-borer .. .. .. 472

4. Mr. Arnott's Operation for Stricture of the Urethra .. .. 474

5. Ectropopharynx, or Œsophagotomy .. .. .. .. .. 475

6. Mr. Shaw on Stricture and Sacculated Bladder .. .. .. 475

### IV. THERAPEUTICS.

1. On Hydrocephalus Acutus, by large Bleedings .. .. .. 479

2. Prevention of Drunkenness .. .. .. .. .. .. 480

3. On the Cholera Morbus of Persia .. .. .. .. .. 481

4. Purpura Hæmorrhagica .. .. .. .. .. .. .. 482

5. Curious Misnomer of Pharmaceutical Agents .. .. .. 482

6. On the Chemical Composition of the Blood during Ptyalism 482

7. Tartrite of Antimony in Large Doses—M. Laennec .. 483

8. Emetine in Violets .. .. .. .. .. .. .. .. 484

9. Injection of Tartar Emetic into the Veins .. .. .. 484

10. On Masked Intermittents .. .. .. .. .. .. .. 485

11. On the Climate, &c. of Madeira .. .. .. .. .. .. 486
12. On the Therapeutical and Physiological Effects of Sulphate of Quinine .. .. .. .. .. .. .. .. .. 487
13. Belladonna preventive of Scarlatina .. .. .. .. .. .. 490

V. MIDWIFERY.

1. Ascites in Pregnancy .. .. .. .. .. .. .. .. 492
2. Cæsarean Operation, successful .. .. .. .. .. 494
3. Nervous Pregnancy .. .. .. .. .. .. .. .. 495
4. Sudden Parturition .. .. .. .. .. .. .. .. .. .. 496
5. The Turn of Life .. .. .. .. .. .. .. .. .. 496
6. Laceration of the Perineum in Parturition .. .. .. 497
7. Rare Case of Complicated Labour .. .. .. .. .. 498
8. Spontaneous Rupture of the Uterus .. .. .. .. .. 499
9. Ascites in Pregnancy (sequel to Art. 1, p. 492) .. .. 500

VI. LEGAL MEDICINE.

1. Wounds by Fire-arms, singular Deception .. .. .. 504
2. Poisoning by Cicuta, with Dissections .. .. .. .. 505
3. The Apothecaries' Act .. .. .. .. .. .. .. .. .. 505
4. Poisoning by Prussic Acid .. .. .. .. .. .. .. 506
5. Simulated Diseases .. .. .. .. .. .. .. .. .. .. 508
6. Medical Remuneration .. .. .. .. .. .. .. .. 509
7. Supposed Punctured Wounds of the Diaphragm .. .. 510
8. Defamation of Character .. .. .. .. .. .. .. 511

XIII.

Bibliographical Record .. .. .. .. .. .. .. .. .. 512

XIV.

EXTRA-LIMITES.

I. Mr. Searle's Instrument for applying Pressure .. .. .. 516
II. Dr. Gordon Smith's Queries .. .. .. .. .. .. .. 519
III. Mr. Battley on the Components of Opium.. .. .. .. 521

XV.

INTELLIGENCE, &c.

I. Society of Physicians of the United Kingdom .. .. .. 523
II. Artificial Seltzer Water .. .. .. .. .. .. .. .. 525
III. Literary Intelligence .. .. .. .. .. .. .. .. 525
IV. Protection of Vaccination.. .. .. .. .. .. .. .. 525

List of Additional Subscribers .. .. .. .. .. .. .. 526

